# Artificial intelligence-based identification of left ventricular systolic dysfunction from 12-lead electrocardiograms: external validation and advanced application of an existing model

**DOI:** 10.1093/ehjdh/ztad081

**Published:** 2023-12-20

**Authors:** Sebastian König, Sven Hohenstein, Anne Nitsche, Vincent Pellissier, Johannes Leiner, Lars Stellmacher, Gerhard Hindricks, Andreas Bollmann

**Affiliations:** Department of Electrophysiology, Heart Center Leipzig at University of Leipzig, Strümpellstr. 39, 04289 Leipzig, Germany; Helios Health Institute, Real World Evidence & Health Technology Assessment, Schwanebecker Chaussee 50, 13125 Berlin, Germany; Helios Health Institute, Real World Evidence & Health Technology Assessment, Schwanebecker Chaussee 50, 13125 Berlin, Germany; Helios Health Institute, Real World Evidence & Health Technology Assessment, Schwanebecker Chaussee 50, 13125 Berlin, Germany; Helios Health Institute, Real World Evidence & Health Technology Assessment, Schwanebecker Chaussee 50, 13125 Berlin, Germany; Department of Electrophysiology, Heart Center Leipzig at University of Leipzig, Strümpellstr. 39, 04289 Leipzig, Germany; Helios Health Institute, Real World Evidence & Health Technology Assessment, Schwanebecker Chaussee 50, 13125 Berlin, Germany; Department of Electrophysiology, Heart Center Leipzig at University of Leipzig, Strümpellstr. 39, 04289 Leipzig, Germany; Helios Health Institute, Real World Evidence & Health Technology Assessment, Schwanebecker Chaussee 50, 13125 Berlin, Germany; Department of Electrophysiology, Heart Center Leipzig at University of Leipzig, Strümpellstr. 39, 04289 Leipzig, Germany; Department of Electrophysiology, Heart Center Leipzig at University of Leipzig, Strümpellstr. 39, 04289 Leipzig, Germany; Helios Health Institute, Real World Evidence & Health Technology Assessment, Schwanebecker Chaussee 50, 13125 Berlin, Germany

**Keywords:** Artificial intelligence, Electrocardiogram, Left ventricular systolic dysfunction, Heart failure

## Abstract

**Aims:**

The diagnostic application of artificial intelligence (AI)-based models to detect cardiovascular diseases from electrocardiograms (ECGs) evolves, and promising results were reported. However, external validation is not available for all published algorithms. The aim of this study was to validate an existing algorithm for the detection of left ventricular systolic dysfunction (LVSD) from 12-lead ECGs.

**Methods and results:**

Patients with digitalized data pairs of 12-lead ECGs and echocardiography (at intervals of ≤7 days) were retrospectively selected from the Heart Center Leipzig ECG and electronic medical records databases. A previously developed AI-based model was applied to ECGs and calculated probabilities for LVSD. The area under the receiver operating characteristic curve (AUROC) was computed overall and in cohorts stratified for baseline and ECG characteristics. Repeated echocardiography studies recorded ≥3 months after index diagnostics were used for follow-up (FU) analysis. At baseline, 42 291 ECG-echocardiography pairs were analysed, and AUROC for LVSD detection was 0.88. Sensitivity and specificity were 82% and 77% for the optimal LVSD probability cut-off based on Youden’s J. AUROCs were lower in ECG subgroups with tachycardia, atrial fibrillation, and wide QRS complexes. In patients without LVSD at baseline and available FU, model-generated high probability for LVSD was associated with a four-fold increased risk of developing LVSD during FU.

**Conclusion:**

We provide the external validation of an existing AI-based ECG-analysing model for the detection of LVSD with robust performance metrics. The association of false positive LVSD screenings at baseline with a deterioration of ventricular function during FU deserves a further evaluation in prospective trials.

## Graphical Abstract

**Graphical Abstract:**
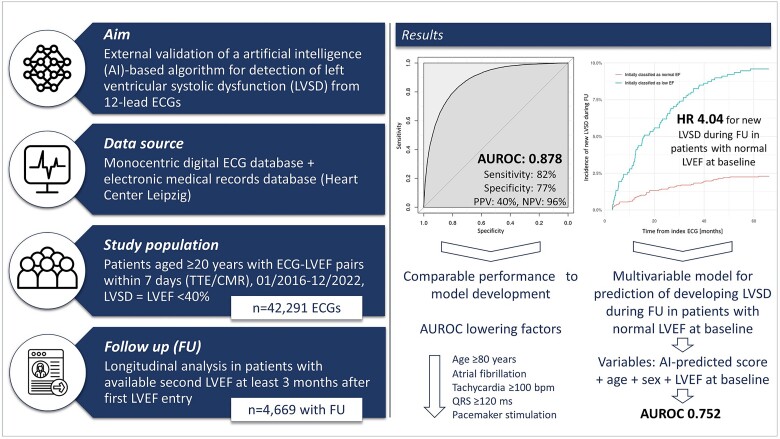

## Introduction

Due to an increasing prevalence over the last years, chronic heart failure (HF) has become one of the most relevant cardiovascular diseases with respect to its medical and socioeconomic impact on health care.^1,2^ An improvement of screening strategies to detect early disease stages and asymptomatic patients has been proposed as an important goal especially in patients with HF and left ventricular systolic dysfunction (LVSD) in order to provide therapies that were shown to improve patients’ outcomes.^3^ To date, neither clinical models nor diagnostic tests are established as regular screening tools for asymptomatic LVSD in Europe. Even the measurement of N-terminal pro-brain natriuretic peptide (NT-proBNP) levels was shown to be of only modest sensitivity.^4^ The advanced analysis of standard surface 12-lead electrocardiograms (ECGs) augmented by artificial intelligence (AI)-based algorithms was introduced as a non-invasive alternative with promising performance measures for the detection of LVSD and superior discriminatory power when compared to NT-proBNP measurement.^5–9^ However, independent external validation is not available for all of the published models.^10,11^ A high variance of performance results for external validation studies reflects both the necessity and difficulty of such analyses, which are influenced by the setting of model application.^12^ Validating an algorithm in an unrelated patient population by a different group of researchers is necessitated to prove its reliability and overcome possible systematic bias.^13^ Yagi *et al*.^14^ published a free-to-use algorithm for the detection of LVSD in 2022, but relevant indicators of model performance as well as values needed for the interpretation and external application were not provided. The model has been developed and tested in populations from North America and Japan with unknown baseline characteristics including an unpublished prevalence of LVSD. Since both patient-related factors and disease prevalence likely influence the prediction of such models, a validation in a European population was considered relevant prior to an utilization in clinical practice.^15,16^ Aims of this study were, therefore, to externally validate an existing algorithm for the detection of LVSD, to describe relevant values and performance metrics in detail, to test strategies for further model improvement, and to investigate whether the model output also predicts future development of LVSD in patients with preserved left ventricular ejection fraction (LVEF) at baseline

## Methods

We performed a retrospective external validation of a previously presented AI-based algorithm for the detection of LVSD from standard surface 12-lead ECGs.^14^ The algorithm was applied to 12-lead ECGs from the monocentric Heart Center Leipzig ECG database recorded between January 2016 and December 2022. Electrocardiograms from both inpatients and patients from outpatient clinics affiliated with the Heart Center Leipzig were included. Only ECGs from patients aged 20 years or older were used according to the selection criteria used in the initial publication. There was no further patient selection (e.g. no exclusion of patients with clinically prevalent HF or known LVSD). Within the Heart Center Leipzig ECG database, ECGs were stored in.xml format with a sampling rate of 500–2000 Hz and a duration of 10.0–20.0 s. All ECGs were down-sampled to 250 Hz and truncated after the first 10 s. Electrocardiograms were written, and automated analysis of digitalized ECG data was performed both by products of Spacelabs Healthcare GmbH (Snoqualmie, WA, USA). Only ECGs with available information on echocardiography-based LVEF were selected for further analyses, and all ECGs of adequate quality based on automated ECG software evaluation were analysed (possible inclusion of multiple ECGs per patient). Echocardiography was performed using a standardized examination protocol and LVEF was assessed either biplane (Simpson’s method) or triplane with LVSD being defined as LVEF < 40% according to the original publication.^14^ Imaging results were extracted from electronic medical records (EMR) and were considered valid only if performed within 7 days from ECG recording. For all patients with a valid ECG-echocardiography pair at baseline, EMR data were searched for follow-up (FU) echocardiography containing LVEF information that was recorded at least 3 months after the first imaging study. All data were anonymized prior to further analysis. There were no missing data.

Electrocardiogram data were applied to a freely accessible (web interface for model application accessible under http://onebraveideaml.org/) convolutional neural network-based model (time required for processing one ECG dataset including data upload: ∼25 s), that is described in more detail in the original publication of Yagi *et al*.^14^ and under at the following URL: https://github.com/obi-ml-public/ECG-LV-Dysfunction. The model’s output is a probability for existing LVSD expressed by a continuous variable between 0 and 1. Artificial intelligence-based ECG analysis was performed both utilizing raw and pre-processed ECG data (https://github.com/PierreElias/IntroECG), the latter implying the elimination of baseline-shifting and outlier voltages in a subgroup of randomly selected ECGs from our overall cohort as described previously.^17^ Considering the use of anonymized clinical routine data, individual informed consent was not obtained. The study was approved by the responsible ethics committee and follows the TRIPOD reporting guidelines for model validation studies.

Model performance was expressed by area under the receiver operating characteristic curve (AUROC, software package used for creation of AUROC graph: https://github.com/overdodactyl/diagnosticSummary/) together with 95% confidence intervals (CIs), sensitivity, specificity, positive predictive value (PPV), and negative predictive value (NPV). Youden’s J was used to define the optimal cut-off for the predicted probability of LVSD, since no cut-off value was published for the application of the original model. We determined the optimal cut-off for maximizing Youden’s J statistic via bootstrapping with 1000 iterations.^18^ In order to avoid overestimation of performance variables and positive bias, we report quality statistics based on out-of-bag samples together with CIs. With this approach, calculations of cut-off point and performance were done with independent datasets in each bootstrap run. Model performance measures were calculated stratified for age, sex, and different ECG parameters that were evaluated by automatic ECG software analysis (heart rate, rhythm, PQ interval, QRS duration, abnormal repolarization). We also tested for the impact of the number of analysed ECGs per patient and the time between ECG recording and imaging study on model’s performance. In patients with available FU echocardiography, the prediction of developing future LVSD in patients with a LVEF ≥ 50% at baseline from index ECGs was tested. For this purpose, the model output (probability of LVSD, included as a logarithmic prediction score) together with additional variables (age, sex, baseline LVEF) were integrated into a multivariable logistic regression model.

## Results

**Figure 1:**
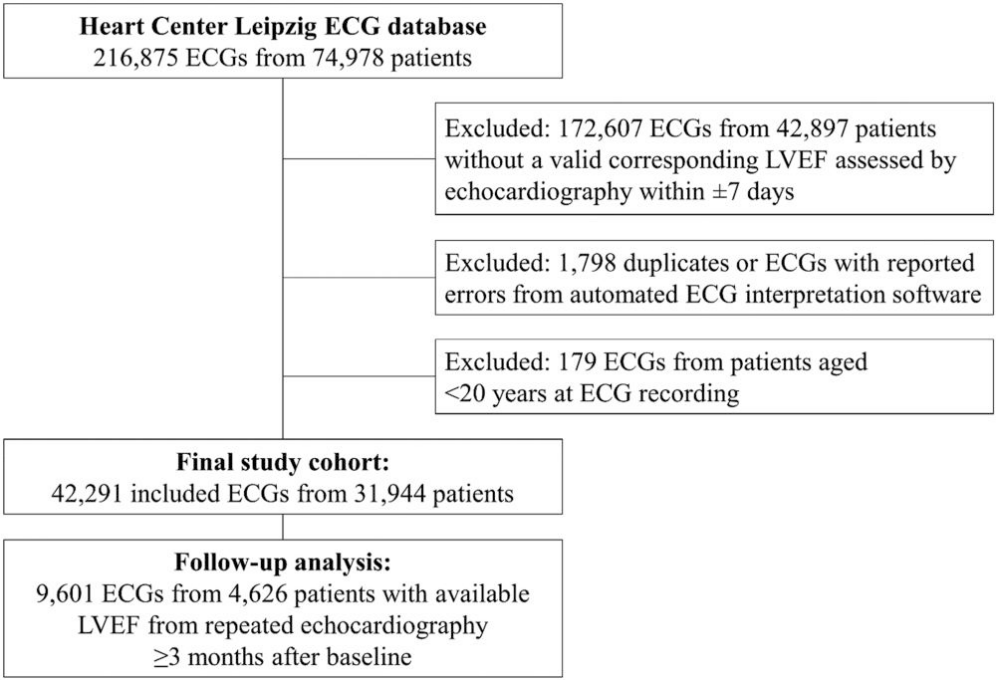
Flow diagram showing the derivation of the study cohort.

**Figure 2:**
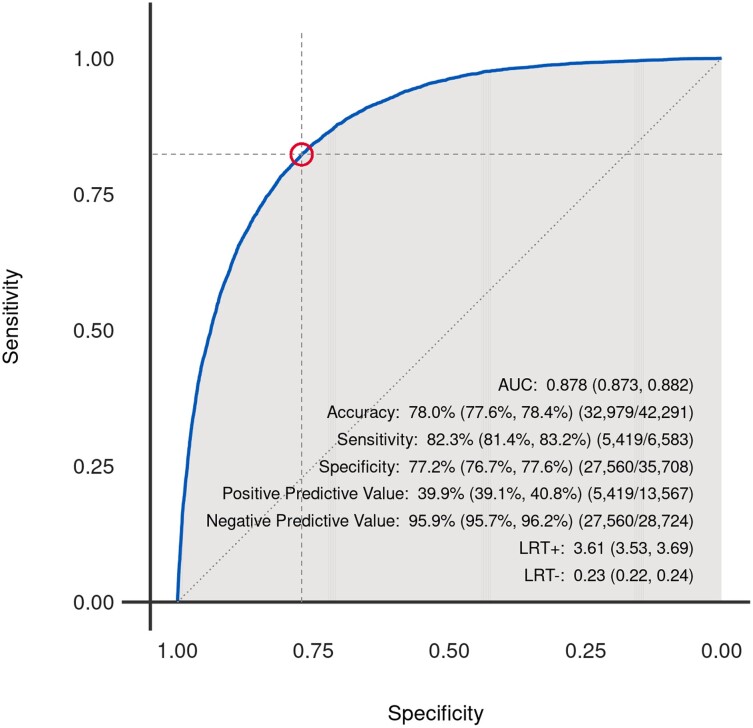
Receiver operating characteristic curve for overall model performance.

**Figure 3:**
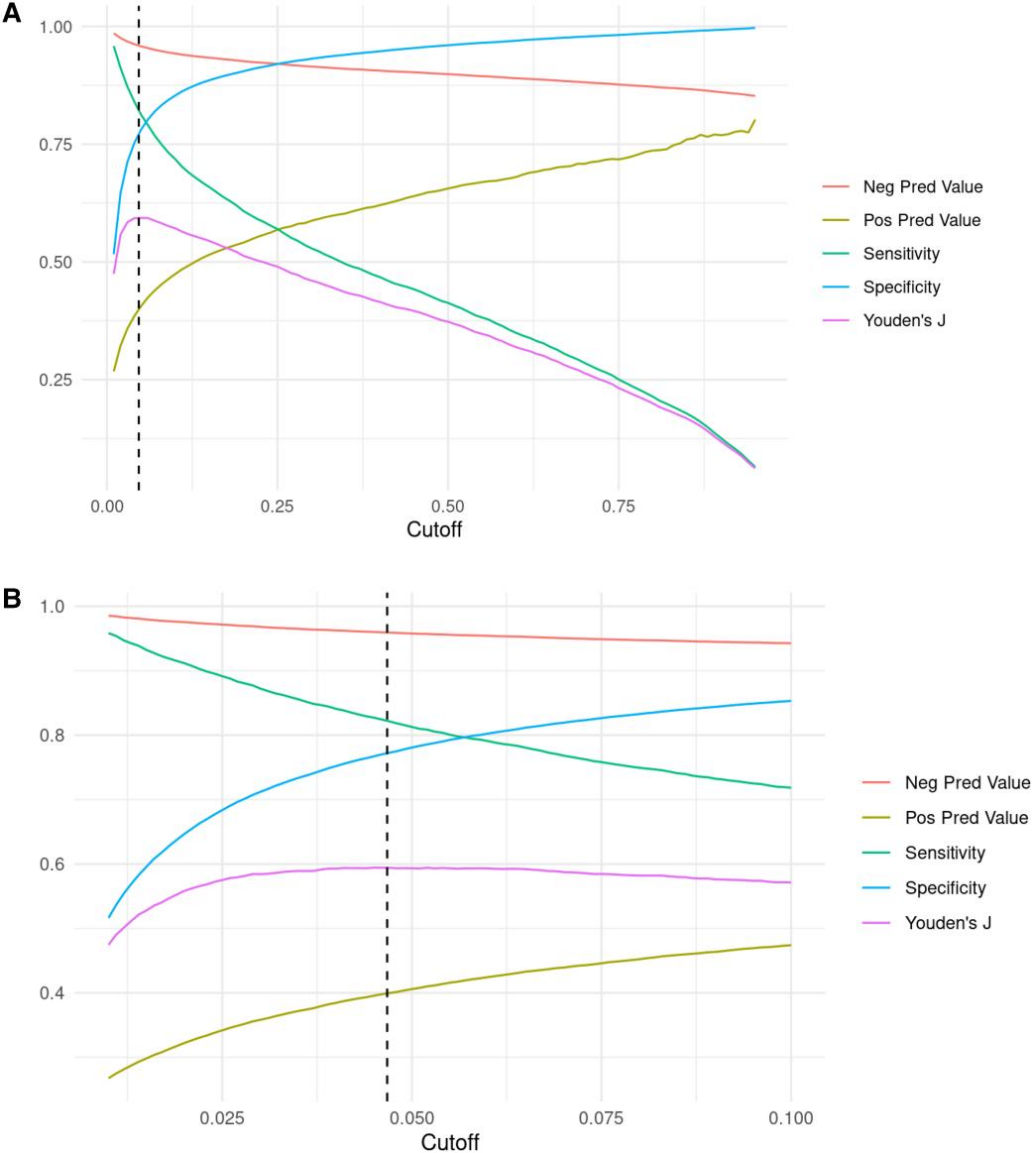
Performance metrics as a function of output probability cut-offs.

**Figure 4:**
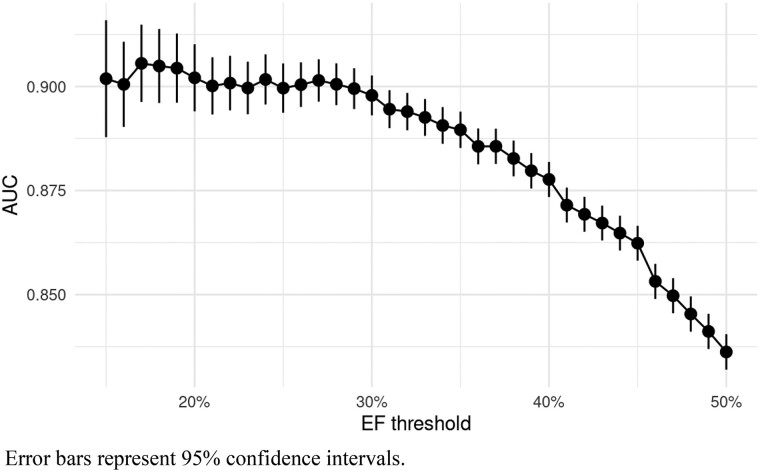
AUROC as a function of LVEF cut-off for the definition of LVSD.

**Figure 5:**
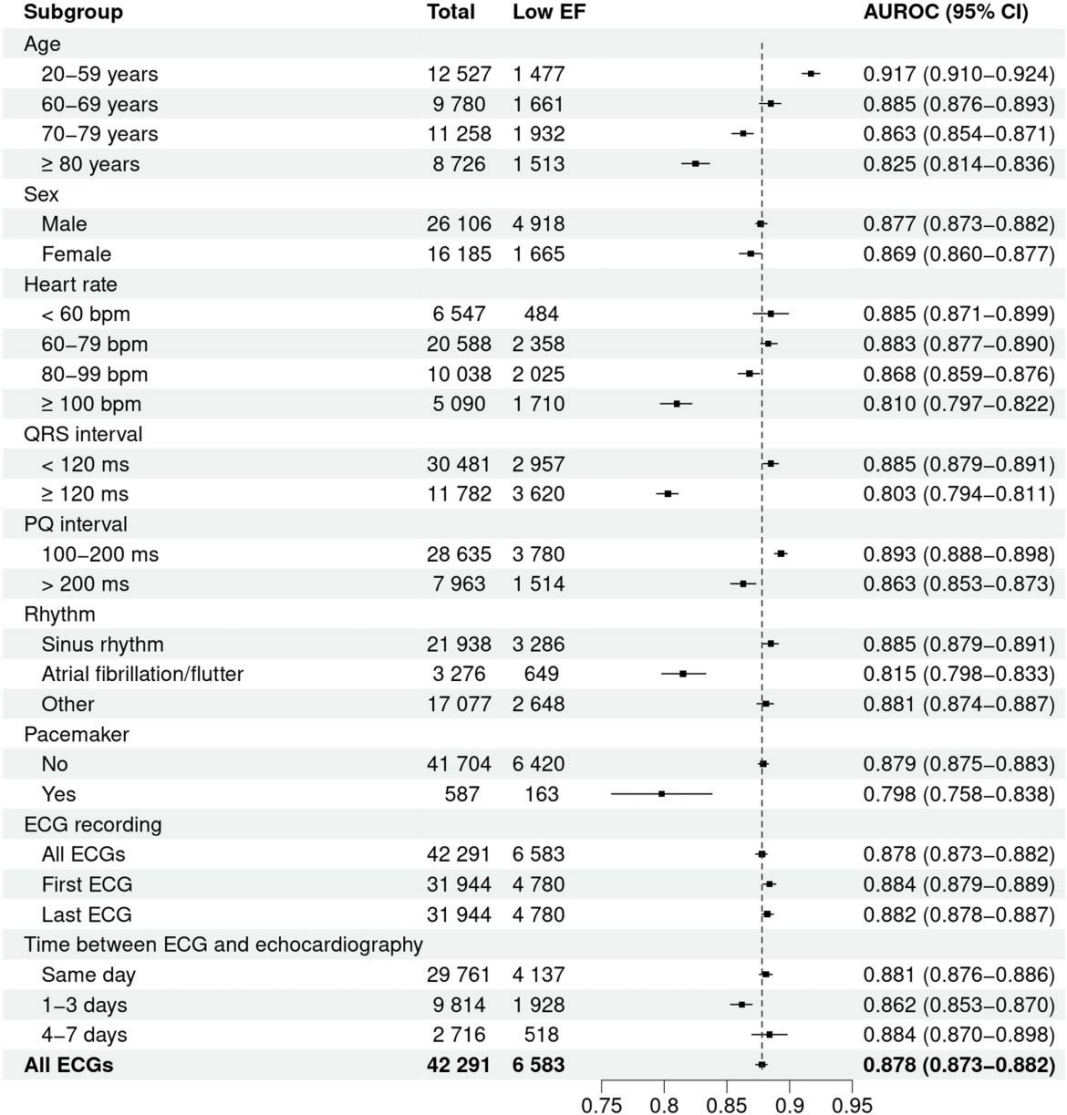
Forrest plot of AUROCs with CIs stratified for baseline and ECG characteristics.

Of 216 875 assessable ECGs, 42 291 valid ECG-echocardiography pairs from 31 944 individual patients were analysed. A flow diagram presenting the derivation of the final study cohort is provided in *Figure 1*. Overall, mean age of cases was 66.2 ± 15.1 years and 38.3% were female. Mean LVEF was 53.3 ± 13.3%, and prevalence of LVSD upon included cases was 14.9%. The AUROC for the original model using non-pre-processed ECG data from our database was 0.88 (95% CI 0.87–0.88; *Figure 2*). Based on Youden’s J, a cut-off of 0.047 (95% CI 0.040–0.064) for the computed probability of LVSD was identified as the best discriminator with a corresponding sensitivity of 82% (95% CI 0.78–0.84), a specificity of 77% (95% CI 0.75–0.81), an accuracy of 78% (95% CI 0.76–0.80), a PPV of 40% (95% CI 0.38–0.43), and a NPV of 96% (95% CI 0.95–0.96) (*Figure 3A* and *B*). Numerical performance measures for the ten model output cut-offs with best Youden’s J values are provided in the Supplementary material. When altering the definition of LVSD as part of an exploratory analysis, calculated AUROCs were 0.89 (95% CI 0.89–0.89) for a LVEF cut-off of <35%, and 0.84 (95% CI 0.83–0.84) for a LVEF cut-off of <50%. Plotting AUROC values for different LVEF thresholds on the *x*-axis, a constantly decreasing curve with a climax in the range between an LVEF cut-off of 15–30% was observed (*Figure 4*). When stratifying for age, sex, and specific ECG parameters, an inferior model accuracy based on AUROC was found in subgroups of patients aged ≥80 years as well as ECGs with heart rate ≥ 100 per minute, wide QRS complexes, present pacemaker stimulation, and present atrial fibrillation/atrial flutter. Neither the time between ECG and echocardiography nor the number and selection of analysed ECGs per patient influenced AUROCs relevantly (*Figure 5*). Electrocardiogram pre-processing was performed in 3185 randomly selected ECGs from 3185 patients (mean age 65.6 ± 15.4 years, 37.7% female, prevalence of LVSD: 14.5%). In this subgroup, there was no difference in model performance when using raw (AUROC 0.89, 95% CI 0.87–0.90) or pre-processed ECG data (AUROC 0.89, 95% CI 0.87–0.90).

**Figure 6:**
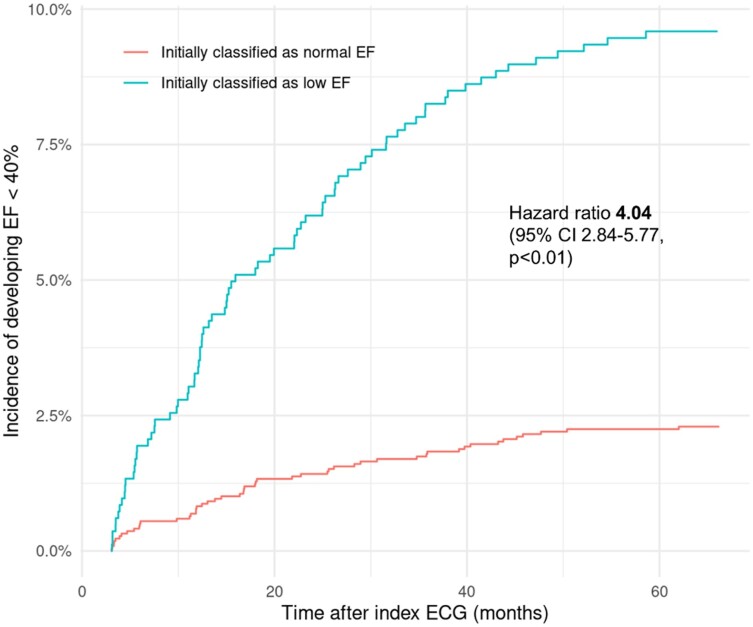
Incidence of LVSD during FU stratified for model output at baseline in patients with initial LVEF ≥ 50%.

In 4669 patients (14.6% of the overall cohort), a FU echocardiography was available (median time from baseline to FU echocardiography 343 days, interquartile range 174–539 days). Within patients with LVEF of ≥50% at baseline (60.7% of patients with available FU data), 4.2% developed LVSD during FU. Applying the AI-based algorithm to corresponding baseline ECGs, a future deterioration of LVEF to <40% was predicted with an AUROC of 0.68 (95% CI 0.63–0.73). The optimal output probability cut-off based on Youden’s J was lower (0.033, 95% CI 0.016–0.045) compared to using the model for LVSD detection at baseline with a resulting sensitivity of 58% (95% CI 0.43–0.68), a specificity of 74% (95% CI 0.60–0.79), a PPV of 9% (95% CI 0.06–0.11), and a NPV of 98% (95% CI 0.97–0.98). An analysis stratifying AUROCs for different patient-related and ECG characteristics is provided in the Supplementary material. Patients with LVEF ≥ 50% at baseline and a high (≥3.3%) predicted probability of LVSD had a more than four-fold increased risk (HR 4.04, 95% CI 2.84–5.77) for developing LVSD during FU compared to patients with a low model-based LVSD probability (*Figure 6*). Integrating the logarithmic AI model output with age, sex, and baseline LVEF into a multivariable model, the AUROC for the prediction of LVSD development during FU was improved to 0.75 (95% CI 0.71–0.79). The AI model-based LVSD probability was an independent predictor for future LVEF deterioration to <40%. Results for odds ratios from univariable and multivariable analyses are summed up in *Table 1*.

**Table 1:** Univariable and multivariable analyses for development of LVSD during FU in patients with normal LVEF at baseline

Variable	Univariable analysis		Multivariable analysis	
	OR (95% CI)	*P* value	OR (95% CI)	*P* value
LVEF at baseline	0.90 (0.87–0.93)	<0.001	0.92 (0.89–0.96)	<0.001
Age	1.75 (1.23–2.55)	0.003	1.74 (1.19–2.59)	0.005
Female sex	0.56 (0.38–0.82)	0.004	0.56 (0.37–0.83)	0.005
Log model output score	1.48 (1.34–1.63)	<0.001	1.37 (1.24–1.52)	<0.001

Odds ratios are given for female compared to male sex, per 25-year increase of age, per per cent increase in LVEF, and per 1 point change of the logarithmic prediction score.

CI, confidence interval; FU, follow-up; LVEF, left ventricular ejection fraction; LVSD, left ventricular systolic dysfunction; OR, odds ratio.

## Discussion

With this retrospective analysis based on the Heart Center Leipzig ECG database, we provide an external validation of an existing AI-based model for the detection of LVSD from standard surface 12-lead ECGs. Applying the model to a different patient population from different continents by an unrelated research team, we were able to show a good model discrimination based on the AUROC that was comparable to the performance metrics published for the development of the original model.^14^ Calculating an optimal cut-off for the model’s outcome probability within our specific population generated an excellent NPV with moderate sensitivity and specificity. The necessity to compute individual cut-offs for patient populations with differing characteristics and a different prevalence of the disease to be detected has been highlighted previously in order to improve results.^19^ A further optimization of model performance through ECG pre-processing was not possible.

To date, there are several published AI-based algorithms for the detection of LVSD with reported AUROCs with a median of 0.90 and a range from 0.84 to 0.95, which is congruent with our results.^10–12,20^ However, not all of those algorithms were truly validated in an external cohort with a differing composition of included subjects when compared to the derivation cohort. This is reflected by a high variability of results for existing external validations. One study showed a decrease in AUROC from 0.93 (model development) to 0.82 (external validation), while others reported even higher AUROCs within their validation cohorts when compared to the model development performance metrics.^19,21,22^ Furthermore, model architecture including used weights and output cut-offs were not made publicly available for all presented models, which hinders comprehensive and independent external validation as well as further model application in clinical practice. Several points have to be considered for the interpretation of those results, like the study-specific prevalence of LVSD, the clinical setting of ECG and echocardiography acquisition, the differing quality and device-based pre-processing of ECGs, a differing assessment and definition of LVSD (quality of echocardiography imaging), and others. One major influencing factor refers to the selection of the patient cohort used for external validation. On the one hand, some AI models were applied to a population that was not related to the derivation cohort at all, whereas, in other studies, validation was performed in relatively homogenous populations being divided only per referring hospitals that were in close proximity to each other.^19,21–23^ Even though there is no further information available on baseline characteristics of the patient cohort used for model development within the original publication, the two cohorts are not related at all and inpatient databases from different continents were used to create them.^14^

With regard to reported sensitivity, specificity, PPV, and NPV, our findings are mid-range when compared to previously published data.^5,6,9,24–26^ The model has a particular value for the exclusion of LVSD as indicated by the high NPV. We decided not to further reduce the LVSD probability threshold in order to achieve an even higher sensitivity. In a primary care setting, it has already been shown that applying a model for LVSD detection with a comparable sensitivity to an unselected patient cohort led to a significantly higher rate of diagnosing heart failure with reduced ejection fraction than the standard-of-care.^27^ A comparable sensitivity can therefore be considered effective with regard to the clinical applicability of the model. Moreover, lowering the cut-off in order to improve sensitivity would also increase the number of false positives and therefore the number of unnecessary tests as a consequence of the model’s output. In this regard, it is important to mention that LVSD prevalence in the population to be tested is of the greatest importance when determining the optimal individual cut-off. Aside from increasing the diagnostic yield, the feasibility of managing cases with potentially identified LVSD in clinical practice as well as the socioeconomic efficiency must also be included in the discussion. Optimizing models’ accuracy would obviously be the best way to reduce the number of false results. Our attempt to improve the model’s discriminatory performance by ECG pre-processing was not successful. A pre-selection of clinical high-risk populations and the integration of available basic clinical information as well as NT-proBNP into combined models may be helpful.^24,25^ Furthermore, confirming the reliability of a model by an external validation in unrelated patient cohorts is indispensable in advance of a broad implementation.

There are other methods of advanced ECG analysis for the detection of LVSD that are not based on AI-based pattern recognition, some of which having been reported to have very good discriminatory power.^28–31^ The comparatively high effort required for data analysis could be on possible reason why such algorithms have not yet been widely used. On the other hand, pathophysiological comprehensibility and transparency are positive aspects. In contrast, the Blackbox character of the above-mentioned AI-based pattern recognition models has to be considered a major limitation of them. However, recently there have been attempts to solve the problem of lacking explainability.^32,33^ Direct comparisons between the two groups of models for the ECG-based detection of LVSD are currently lacking.

When stratifying results for age, sex, and several ECG parameters, we confirmed the findings provided by Yagi *et al*.^14^ with lower AUROCs in octogenarians, prevalent atrial fibrillation and ECGs with wide QRS complexes. This is in line with findings from other working groups and models.^26,34,35^ Since the clinical diagnoses of atrial fibrillation or existing (left) bundle branch block are likely to be considered as potential indicators of structural heart disease by clinicians, an echocardiography will be performed in most cases either way. Therefore, the inferior discriminatory power of AI models related to these ECG patterns should not be relevant in the context of population screening for LVSD. Rather, it can and should be discussed whether separate algorithms should be developed for patients with and without such obvious electrical abnormalities like complete bundle branch block or atrial fibrillation in order to further improve the models’ respective predictions. The fact that AUROCs were lower when definitions of LVSD also included cases with only mildly reduced LVEF has also been shown previously.^23,36^ Of note, we were able to show that patients with a preserved LVEF and a false positive AI-based result for LVSD detection from baseline ECG were at an increased risk for the development of an impaired LVEF during FU compared to patients with a low AI model-computed LVSD probability. Similar observations were made for the model that was presented by colleagues from the Mayo clinic and another recently presented model from Taiwan.^6,25,37^ Of course, all models were not developed for the future detection of LVSD in patients without impaired LVEF at baseline, which might explain the inferior AUROC for this outcome as shown in our analysis. However, even though the performance metrics were lower when compared to corresponding results for LVSD detection at baseline, the huge difference with regard to incidences of LVSD over time has to be considered clinically meaningful. Moreover, after integrating the model’s output (probability of LVSD) at baseline with age, sex, and baseline ejection fraction into a multivariable model, the AUROC improved to 0.75, which could definitely serve as a starting point for an intensified clinical FU. In this light, Chen *et al*.^12^ reported increased major adverse cardiovascular event rates in patients with a high predicted probability of LVSD despite preserved LVEF at baseline. Further research is needed to assess the additional value of AI-based ECG analysis for the screening of LVSD at baseline and during FU from a clinical and socioeconomical perspective.

## Limitations

There are several limitations related to this study. First of all, the technical background of ECG recording as well as the quality of ECG data may be different from the original model development study, which could have influenced results. Due to the sample size of our cohort, a manual quality check of included ECGs was not possible and quality assurance relied on an automated software algorithm for the detection of ECGs with unacceptable quality. Moreover, there was no information on the clinical setting of ECG assessment from the development study by Yagi and colleagues. However, proving the reliability and reproducibility of the model’s performance in different patient populations and across different technical requirements is a major goal of external validation. The cut-off value for model output probabilities that was used within the data analyses presented in the original study was not made available by Yagi and colleagues. Therefore, we had to compute an individual cut-off probability with optimized model performance metrics based on our validation dataset. This hinders direct comparability of all described performance measures and is a major limitation. To enable an external validation and further application of an AI-based prediction model, it is of outmost importance to publish the model’s architecture including used weights and cut-off values for model’s output.

Due to data availability, we were not able to validate the model with prospectively collected data to further add information on reliability and usability as a prediction tool for clinical practice. Furthermore, electronic data on NT-proBNP were available only for a minority of patients from our ECG database, which is why we were not able to compare predictive performance of the examined AI model and NT-proBNP for the detection of LVSD. With regard to the stratification of performance metrics according to different ECG patterns, we relied on the automatic software analysis of ECGs for the detection of atrial fibrillation, wide QRS complexes, and other variables. This carries an unquantifiable risk of misinterpretation, but was unavoidable due to the size of the database. Second, the mode of LVEF assessment from the original study was not described in further detail, which hinders a comparison with our data. Moreover, there may be inconsistencies within our EMR database with respect to LVEF values as they were collected retrospectively and not stored for research purposes. There was no re-evaluation of echocardiography findings. Lastly, there was no regular and scheduled FU for all patients including repeated echocardiography. This is central to the interpretation of LVSD prediction during FU in patients without LVEF impairment at baseline and may have influenced results. A prospective evaluation with planned FU imaging is required to generate more valid data in this regard.

## Conclusion

With this study, we provide the external validation of an existing AI-based ECG-analysing model for the detection of LVSD with excellent and robust performance metrics. Several ECG patterns that influenced the model’s discrimination were identified. Moreover, patients with preserved LVEF but model-generated increased probability of LVSD at baseline were shown to be at higher risk for a future deterioration of LVSD, which deserves a further evaluation in prospective trials.

## Supplementary material

Supplementary material is available at *European Heart Journal – Digital Health*.

## Supplementary Material

ztad081_Supplementary_Data

## Data Availability

The data underlying this article will be shared on reasonable request to the corresponding author.

## Permissions information

The authors do hereby declare that all illustrations and figures in the manuscript are entirely original and do not require reprint permission.
