# Supplementary material for: Artificial intelligence-based identification of left ventricular systolic dysfunction from 12-lead electrocardiograms: external validation and advanced application of an existing model
Source: Eur Heart J Digit Health. 2023 Dec 20;5(2):144–51. doi: 10.1093/ehjdh/ztad081 (PMC10944686; doi:10.1093/ehjdh/ztad081)

**Supplementary material**

Supplemental Table 1: Model performance measures for different output probability cutoffs

Supplemental Figure 1: Forrest plot of AUROCs for the prediction of incident LVSD during FU with CIs stratified for baseline and ECG characteristics

**Supplemental Table 1: Model performance measures for different output probability cutoffs**

| Cutoff | Sensitivity | Specificity | PPV | NPV | F1 | Accuracy | Youden’s J |
| --- | --- | --- | --- | --- | --- | --- | --- |
| 0.01 | 96% | 52% | 27% | 99% | 0.42 | 59% | 0.475 |
| 0.02 | 91% | 65% | 32% | 98% | 0.48 | 69% | 0.558 |
| 0.03 | 87% | 71% | 36% | 97% | 0.51 | 74% | 0.584 |
| 0.04 | 84% | 75% | 38% | 96% | 0.53 | 77% | 0.593 |
| 0.047^*^ | 82% | 77% | 40% | 96% | 0.54 | 78% | 0.595 |
| 0.05 | 81% | 78% | 41% | 96% | 0.54 | 79% | 0.593 |
| 0.10 | 72% | 85% | 47% | 94% | 0.57 | 83% | 0.571 |
| 0.15 | 66% | 89% | 52% | 93% | 0.58 | 85% | 0.545 |
| 0.20 | 61% | 90% | 54% | 93% | 0.57 | 86% | 0.513 |
| 0.25 | 57% | 92% | 57% | 92% | 0.57 | 87% | 0.490 |

^*^ Identified as best cutoff based on Youden’s J.

NPV: Negative predictive value; PPV: Positive predictive value

**Supplemental Figure 1: Forrest plot of AUROCs for the prediction of incident LVSD during FU with CIs stratified for baseline and ECG characteristics**


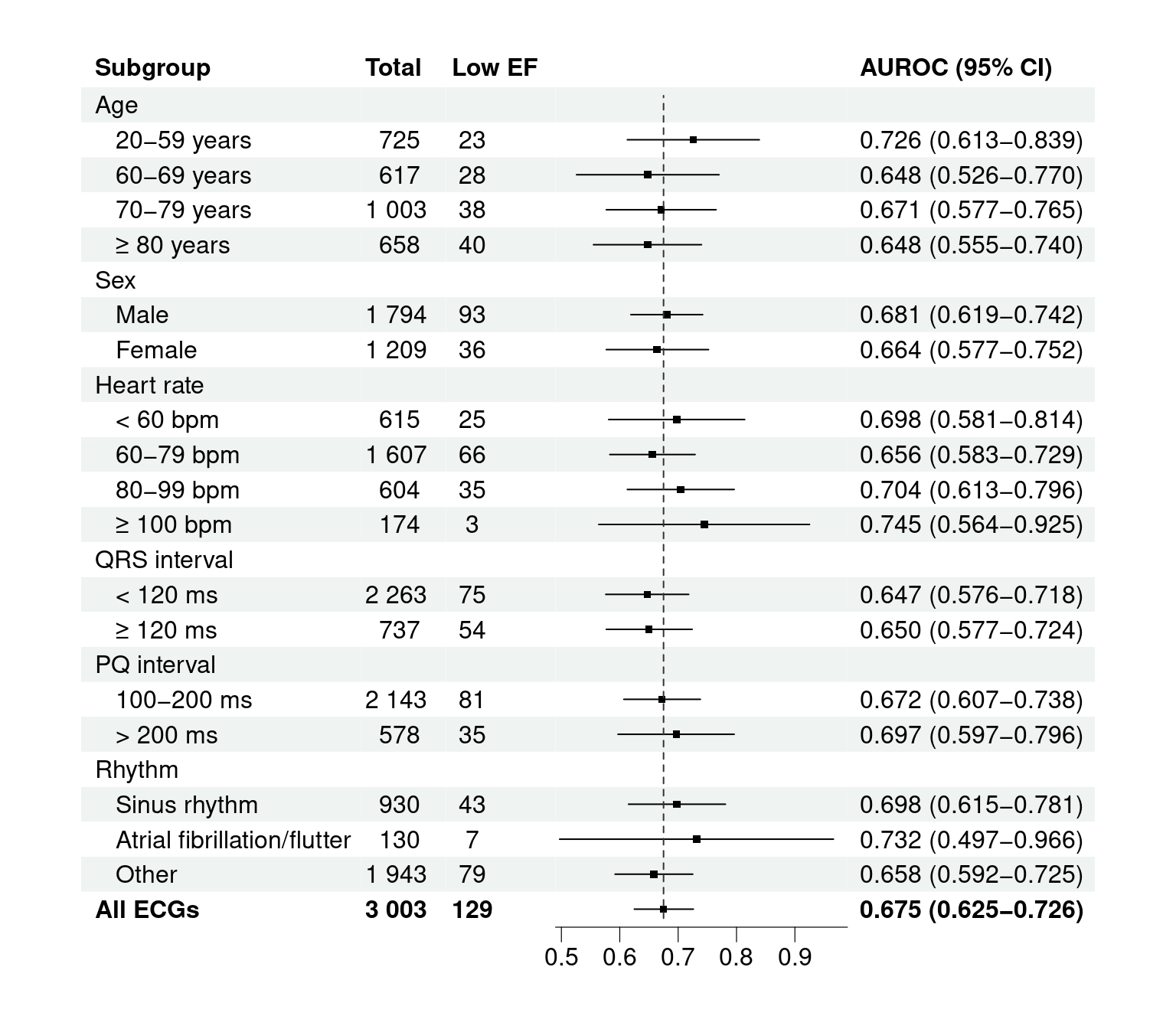

Supplement: ztad081_Supplementary_Data [file ztad081_supplementary_data.docx]
